# Contribution of the Type II Chaperonin, TRiC/CCT, to Oncogenesis

**DOI:** 10.3390/ijms161125975

**Published:** 2015-11-06

**Authors:** Soung-Hun Roh, Moses Kasembeli, Deenadayalan Bakthavatsalam, Wah Chiu, David J. Tweardy

**Affiliations:** 1 Department of Biochemistry and Molecular Biology, Baylor College of Medicine, Houston, TX 77030, USA; sroh@bcm.edu (S.-H.R.); wah@bcm.edu (W.C.); 2 Division of Internal Medicine, the University of Texas MD Anderson Cancer Center, Houston, TX 77030, USA; mmkasembeli@mdanderson.org; 3 Department of Molecular Cardiology, Texas Heart Institute, Houston, TX 77030, USA; dbakthavatsalam@texasheart.org

**Keywords:** protein-folding, proteostasis, oncogenesis, chaperone, chaperonin, HSP70/90, TRiC/CCT, oncoprotein

## Abstract

The folding of newly synthesized proteins and the maintenance of pre-existing proteins are essential in sustaining a living cell. A network of molecular chaperones tightly guides the folding, intracellular localization, and proteolytic turnover of proteins. Many of the key regulators of cell growth and differentiation have been identified as clients of molecular chaperones, which implies that chaperones are potential mediators of oncogenesis. In this review, we briefly provide an overview of the role of chaperones, including HSP70 and HSP90, in cancer. We further summarize and highlight the emerging the role of chaperonin TRiC (T-complex protein-1 ring complex, also known as CCT) in the development and progression of cancer mediated through its critical interactions with oncogenic clients that modulate growth deregulation, apoptosis, and genome instability in cancer cells. Elucidation of how TRiC modulates the folding and function of oncogenic clients will provide strategies for developing novel cancer therapies.

## 1. Molecular Chaperones and Oncogenesis

**Figure 1:**
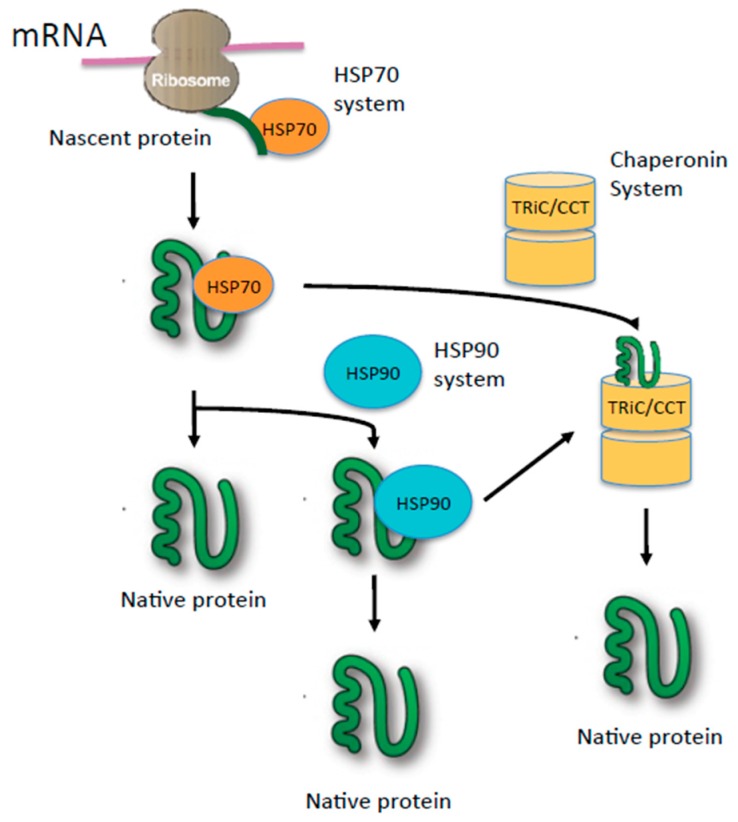
Model of the chaperone network involving HSP70, HSP90, and TRiC (T-complex protein-1 ring complex, also known as CCT). Newly synthesized nascent polypeptide chain interacts with the HSP70 family, which mediates folding either co-translationally or post-translationally. HSP70 also can deliver a folding-intermediate to downstream chaperones HSP90 or TRiC/CCT to complete folding.

Molecular chaperones constitute a major arm of the proteostasis network (Figure 1); they play a central role in the maintenance of protein homeostasis through an intricate system of cooperative mechanisms that balance protein biosynthesis, folding, translocation, assembly/disassembly, and clearance [1,2]. Molecular chaperones are a diverse group of proteins that interact with and assist other proteins to properly attain functional conformation [3]. They can be classified into two mechanistic classes—chaperones that promote folding of non-native proteins by binding to and releasing their substrates into the bulk matrix of the cell and chaperones that promote folding by sequestering single protein molecules within a molecular cage (chaperonins). The former include most heat shock proteins (HSPs) whose expression is known to be up regulated in response to environmental stress, most prominently, HSP70 and HSP90. The eukaryotic chaperonin family includes the type I chaperonin, HSP60, and the type II hetero-oligomeric chaperonin, TRiC (T-complex protein-1 ring complex, also known as CCT). Chaperones often function as large protein complexes that include other proteins called co-chaperones. They are essential for cell survival as they protect against proteotoxic stress that may lead to protein misfolding and aggregation. The primary action of chaperones is to transiently bind to hydrophobic regions of nascent or stress denatured proteins and prevent aggregation during the folding process [4].

Increased protein chaperone capacity has been linked to the etiology of many diseases including cancer [5]. Considerable evidence now exists implicating molecular chaperones in the development of cancer; they have been shown to play a fundamental role in the molecular mechanisms that lead to the emergence and progression of the tumor phenotype. Accordingly, levels of heat-shock factor 1 (HSF1), a transcriptional regulator of protein homeostasis that activates the transcription of HSPs most importantly HSP70 and HSP90, has been shown to be elevated in many cancer cell lines and tumors [6]. Indeed, the expression of several HSPs is increased in many tumors, such that HSPs are prognostic biomarkers in cancer and their overexpression is linked to poor survival and response to therapy [7]. The sensitivity of a wide variety of tumors to inhibitors of HSP90 and HSP70 exemplifies the importance of molecular chaperones in maintaining survival of cancer cells [8,9].

The contribution of TRiC to cancer has not received as much attention as members of HSPs [5,7,9]; however, evidence is now emerging implicating TRiC in the pathogenesis of numerous cancers. Importantly, several proteins associated with tumorigenesis have been identified as *bona fide* TRiC clients; these include signal transducer and activator transcription (STAT3), cyclins B and E, P53 and Von Hippel-Lindau [10,11,12,13,14].

A recent study suggested that TRiC subunits, CCT2 and CCT1, are essential for survival and proliferation of breast cancer [15]. CCT1 was shown to be transcriptionally modulated by the driver oncogene, phosphatidylinositide 3-kinases (PI3K). Whether these observations reflect the protein folding function of TRiC complex or a non-chaperoning role of individual subunits is not clear; however, individual subunits of TRiC have been shown to have protein-folding capacity [16]. While higher expression levels of TRiC have been associated with tumorigenesis, a recent analysis of cancer cell lines appeared to show less correlation between TRiC concentrations and its specific activity The disparity between TRiC concentrations and TRiC activity has been attributed to the dynamic partitioning of substrates between TRiC, its co-chaperones, and HSPs that seems to be influenced by concentrations of HSP70 within the cell [8,17].

## 2. Role of Heat Shock Proteins (HSPs) in Oncogenic Signaling

Chaperone support and maintenance of oncogenic signaling pathways is integral to the biology of malignant tumor initiation and growth. For example, HSP90’s activity is necessary for the conformational stability and activity of many kinases, transcription factors and hormones most of which are known oncogenes or closely linked to oncogenic signaling pathways [18,19,20]. The cytoprotective qualities of the protein folding network and its ability to adaptively respond to environmental cues have been co-opted in cancer. Chaperones are essential in supporting events that induce malignant cell transformation such as mutations or increased expression of oncogenic proteins and play a crucial role in helping rewire signaling pathways and networks toward increased survival and proliferation as well as increased immune evasion [5,9]. Because of the universal role that chaperones play in supporting cellular signaling networks, it is not surprising that they have been implicated in all characteristic hallmarks of cancer, *i.e.*, self-sufficiency in growth signals, insensitivity to growth-inhibitory signals, evasion of apoptosis and increased replicative potential [21]. It is clear that the action of chaperones underpins molecular aspects of these hallmarks. For example, both HSP70s and HSP90 are known to have a critical role in the modulation of programmed cell death (PCD) and the ability of cancer cells to avoid apoptosis. HSP90 and HSP70 help cancer cells avoid apoptosis by the direct modulation of the apoptotic machinery or by controlling the activation signaling networks that control apoptosis [22]. Interestingly, TRiC also associates with STAT3 a transcriptional regulator of anti-apoptotic proteins [10].

Cellular identity is mainly a product of epigenetic regulation. The modulators of epigenetic changes are increasingly being accepted as important drivers of the cancer phenotype. Thus, in addition to buffering genomic alterations during oncogenesis, chaperones, especially HSP90, have been implicated in the direct modulation of the epigenetic memory system and transcription regulation [23,24].

Perhaps more significantly is the role of chaperones within the tumor microenvironment. The tumor is a harsh environment for cellular life; in order for cells to survive they must be able to withstand adverse conditions, such as low oxygen levels, low pH and lack of nutrients. Chaperones have been shown to play a critical role in the adaptive mechanisms within the hostile tumor microenvironment. Induction of tumor angiogenesis is one of the critical adaptations necessary to overcome oxygen and nutrient deficiencies and to promote tumor growth and progression. Hypoxia-inducible factor-1α (HIF-1α), the extremely labile master regulator of angiogenesis, is stabilized by HSP70 and HSP90 [25]. HSP70 and TRiC are required for the proper folding of Von Hippel-Lindau protein that modulates, in an oxygen-dependent manner, the concentration of hypoxia-inducible transcription factor, HIF-1α, and other proteins required for tumor growth and vascularization [14,26].

As discussed previously, it has been hypothesized that chaperones such as HSP90 cushion the impact of genomic mutations, thus facilitating the evolutionary process [27,28]. Oncogenesis in itself can be looked upon as a micro-evolutionary process in which the hostile environment within the tumor provides selection pressure [29]. Indeed, it is becoming increasingly evident that elevated chaperone activity imparts a survival advantage on transformed cells over normal non-transformed ones within the tumor microenvironment. The adverse tumor conditions have been shown to be a major source of DNA damage, mutagenesis and genetic instability that lead to more transformation, tumor heterogeneity and drug resistance [30,31]. In this review, we highlight the role of chaperones especially chaperonin TRiC/CCT in tumorigenesis and discuss recent findings looking at its impact on the folding and function of oncoproteins as well as tumor suppressors.

## 3. TRiC: The Protein-Folding Machine in Eukaryotes

**Figure 2:**
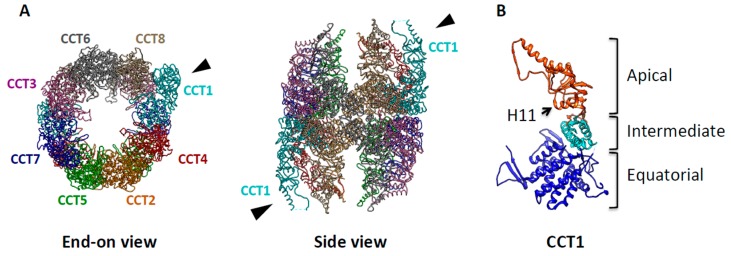
Molecular architecture of TRiC/CCT. (**A**) The end-on and side views of the X-ray crystal structure of TRiC, modified from PDB ID 2XSM, show that TRiC is a double-ringed structure composed of eight homologous but distinct subunits (CCT1–8). Each subunit is shown having a different color with the specific subunit order indicated. The black arrowhead indicates CCT1, which has unique asymmetrical features; (**B**) The X-ray structure of CCT1 is shown as a representative subunit. Helix-11 (H11), the putative substrate recognition site is indicated by the arrow.

TRiC (T-complex protein-1 ring complex, also known as CCT) is an essential 1 MDa eukaryotic chaperonin. It has a double-ring structure with a central cavity in each ring [32]; each ring is composed of eight homologous but distinct subunits (CCT 1–8) [33,34], arranged in a specific order [35,36] (Figure 2A). Each subunit is ~60 kDa and consists of three domains—apical, intermediate, and equatorial (Figure 2B). While the sequence of the equatorial and intermediate domains is conserved, that of the apical domains is highly diverged among the eight subunits [37]. Multiple structures have been reported for TRiC [38,39,40] using x-ray crystallography (Figure 2A) and single particle cryo-EM.

TRiC assists productive folding of substrate proteins by undergoing conformational changes that are ATP-dependent [41,42,43,44]. The structures of the TRiC in the presence of varied nucleotide conditions have been solved using cryo-EM at intermediate resolution, which have lead to an improved understanding of the large conformational changes that occur upon nucleotide binding and hydrolysis [40,45,46]. The conformational cycling begins with the binding of ATP and a transition of the complex to the closed conformation required for ATP hydrolysis to bring the lid helices into close proximity. Opening of the lid occurs in conjunction with releasing ADP from the active site. The complex can exist in an asymmetrical conformation with one ring closed and one open even during ATP cycling conditions, suggesting a inter-ring allosteric model mediated through a two-stroke mechanism [47]. However, the allosteric communication that occurs between the rings is not well understood.

Although the mechanisms of TRiC-substrate recognition, binding, and folding remain under investigation, each of the subunits can recognize different polar and hydrophobic motifs within substrate proteins [48]. TRiC interacts with approximately 10% of the proteome and its function is absolutely essential for viability [49]. TRiC substrates have been identified by multiple groups [50,51] and, recently, Yam and colleagues determined ~200 cellular TRiC substrates using proteomic global approaches based on immunoprecipitation and mass spectroscopy [49]. From the secondary structure analysis of the substrates, TRiC has been shown to mediate the folding of a number of β-sheet rich proteins, including telomerase cofactor TCAB1 [52], the cell cycle regulators CDC20 and CDH1 [49], as well as members of the STAT family of transcription factors [10]. However, the full repertoire of TRiC substrates remains underdetermined, in part, because specific motifs characteristic of TRiC substrates have not been identified. As a general principle, TRiC substrates have a higher potential to aggregate. Substrate proteins also are often large, have extended hydrophobic stretches, or are involved in multi-protein complexes [53]. The ability to assist such a wide range of proteins with diverse folds and sequence properties raises the potential for multiple mechanisms through which TRiC can recognize substrates and promote their folding.

Recent *in vitro* work has illuminated the molecular determinants of substrate interaction with the apical domains of TRiC subunits. Using known subunit-substrate pairs, Joachimiak and colleagues have demonstrated that substrate motifs are recognized by a cleft formed between Helix 11 (H11) and a proximal loop in the TRiC apical domains (Figure 2B) [48]. In addition, the subunit arrangement recently has been identified with a combinatorial approach that employed X-ray crystallography and chemical crosslinking mass spectrometry as well as bioinformatics [35,36,54] (Figure 2A). The subunit arrangement of TRiC leads to the spatial partitioning of subunits with different chemical properties. Specifically, subunits are segregated by their ATP-binding affinities (High affinity subunit: CCT1,2,4,5, Low affinity subunit: CCT3,6,7,8) [55]. Thus, the hetero-oligomeric nature of TRiC generates chemically asymmetric features, which likely provide the basis for the unique ability of TRiC to fold specific substrates [56,57]. The meaning of this asymmetric nucleotide usage and allosteric mechanism in the context of protein folding remains an area of ongoing research [58].

## 4. TRiC Binds to and Modulates Cancer Related Proteins

TRiC has been estimated to directly assist the folding of as many as 10% of cytosolic proteins [49,59] and it provides the unique ability to fold certain proteins that cannot be folded by simpler chaperone systems. This strict requirement of TRiC is also essential for folding proteins involved in oncogenesis. This suggests that TRiC plays a potential role in cancer cell development by direct modulation of the folding and activity for client proteins related to oncogenesis, such as tumor suppressor Von Hippel-Lindau (VHL) [14,60] and p53 [12,61] as well as the pro-oncogenic protein STAT3 [10].

### 4.1. Chaperonin TRiC Works as an Assembly Station for the Tumor Suppressor, VHL (Von Hippel-Lindau)

Adaptation to environmental changes in oxygen levels is essential to the survival of all eukaryotic cells. Most eukaryotic cells have evolved mechanisms to monitor changes in O_2_ levels and adaptively mount a homeostatic gene regulation response that modulates key cellular functions such as glucose transport, metabolism and angiogenesis [62]. It is well established that deregulation of the oxygen sensing pathway leads to the development and maintenance of the tumorigenic state; as such, tumor hypoxia and dysregulated metabolism are salient features of cancer. Central to the adaptive mechanism of the hypoxia response pathway is the tumor suppressor, E3 ligase, Von Hippel-Lindau protein (VHL), which plays a key part in cellular oxygen sensing by targeting hypoxia-inducible factors (HIF) for proteosomal degradation. Mutations that inactivate VHL protein have been associated with a variety of tumor systems including clear-cell renal-cell carcinoma, pheochromocytoma, pancreatic tumors, central nervous system, and retinal hemangioblastoma [63,64]. VHL also is inactivated in approximately 80% of sporadic renal cell carcinomas, the most common form of kidney cancer [13,65,66]. VHL’s function as tumor suppressor requires its association with a ubiquitous complex composed of two small proteins, Elongin B and Elongin C, via a linear sequence termed the “BC box,” comprising amino acids 157–172 [67,68,69]. This complex functions as part of an SCF-like ubiquitin-ligase that promotes the destruction of target proteins required for growth and vascularization of solid tumors [70,71,72]. The best-known substrate of the VCB complex is HIF-1α, which is involved in cell response to oxygen levels [73,74,75].

No crystal structure of unbound VHL is currently available, probably due to its molten globule conformation [76]. Therefore, most of the structural data regarding VHL has been derived from X-ray crystallography of a 19-kDa portion of VHL associated with Elongin B and C [77]. Frydman and colleagues have shown that VHL associates with TRiC and that the chaperonin is required for the ATP-dependent formation of the VBC complex (VHL, Elongin B and C). Release of VHL from TRiC requires VHL binding to Elongin B and C and is impaired by tumor-producing mutations that affect the BC-binding site [26]. Both HSP70 and the chaperonin TRiC are required for correct folding of newly translated VHL, which is coupled to assembly of the VBC complex [60,78]. HSP70 and TRiC appear to function sequentially in the VHL folding pathway, with loss of HSP70 function blocking association with TRiC and loss of TRiC function having no effect on HSP70 association [14]. Furthermore, the TRiC-binding site of VHL was defined as a 55 amino-acid region that corresponds closely to exon 2 of VHL. This region is both necessary and sufficient for chaperonin binding, and its loss, which leads to tumor formation, abrogates formation of a correctly folded VBC complex. It is now well established that disease causing mutations of VHL disrupt the chaperone pathway leading to rapid degradation of VHL, thus inactivating the E3 ligase activity towards HIF proteins [26]. In the absence of VHL activity, HIF transcriptional activity is constitutively active. A wide variety of human cancers have been shown to elevate HIF protein levels [79]. In fact there is strong evidence showing that unconstrained HIF transcriptional activity plays an important role in tumor development and has been implicated in many of the key hallmarks of cancer including, angiogenesis, metabolic dysregulation, proliferation, and metastasis [80].

In addition to VHL, immunoprecipitation and mass spectrometry studies indicate that prolyl hydroxylase domain 3 (PHD3) is a substrate of TRiC [81]. Members of the PHD family of hydroxylases serve as important sensors of hypoxia; they are involved in tuning cellular responses to oxygen levels. There are three known PHDs that modulate HIF1 activity in mammalian cells, PHD1, PHD2, and PHD3 [82]. In the presence of oxygen, PHDs mark HIF1 for ubiquitination and subsequent degradation by hydroxylating key proline residues within HIF1. Conversely low O_2_ levels inactivate PHDs leading to increased levels of HIF1 and transcription of downstream target genes that mediate the adaptive responses to hypoxia [83]. Several studies show that PHD3 is overexpressed in pancreatic cancer and might influence patient survival [84], although, these observations appear paradoxical in the sense that increased levels of PHD activity have tumor suppressor function, it remains clear that elevated PHD3 is associated with aggressive pancreatic cancer and poor patient outcome [85]. Thus, further studies are necessary to delineate the role that PHD3 plays in tumor growth. The consequences of TRiC’s interaction with PHD, in relation to TRiC’s tumor promotion function, also merit additional investigation. Collectively these observations support the hypothesis that TRiC and HSP chaperones play an important role in maintaining oxygen homeostasis and that impaired proteostasis of oxygen sensing pathways is a major contributing factor to carcinogenesis.

### 4.2. TRiC Contributes to STAT Protein Folding and Function

It has long been known that chaperones are essential for the maintenance of signaling pathways that promote tumorigenesis. For instance, chaperones such as HSP90 are required for the stabilization and maturation of nuclear hormone receptors, transcription factors, and protein kinases that are commonly dysregulated during tumorigenesis [86]. We have previously reviewed the role of chaperones in JAK/STAT signaling and its implications to cancer and other diseases [87]. Signal transducer and activator of transcription 3 (STAT3) is a sequence-specific DNA-binding transcription factor responsible for the transmission of peptide hormone signals from receptors on the extracellular membrane to nucleus. There is clear evidence that STAT proteins are dependent on chaperones to function properly. Several groups have shown a physical association between chaperones and STAT1, 3, and 5. HSP90 inhibitors have been shown to reduce levels of total and phosphorylated STAT3 in ANBL-5 and ANA-6 myeloma cell lines [88]. It is hypothesized that HSP90 and HSP70 modulate STAT activity by stabilizing the active conformation of phosphorylated dimers [89]. We demonstrated recently that the chaperonin TRiC modulates the folding and function of STAT3 [10]. Constitutive activation of STAT3 is a common feature of many solid and hematologic tumors [90]. Aberrant activation of STAT3 has been shown to have potent oncogenic properties [91] as it plays a central role in the regulation of many transcriptional programs involved in tumorigenesis, including those that promote cell survival, cell cycle progression and angiogenesis [92]. In addition dysregulated STAT3 signaling has also been shown to promote tumor growth and metastasis, in part, through suppression of antitumor innate and adaptive immune responses [93]. Our data indicates that STAT3 is a *bona fide* substrate of TRiC. We showed that STAT3 requires TRiC for folding and proper functioning. We showed that genetic targeting of TRiC in HS-578T and HEPG2 cancer cell lines, known to have constitutive STAT3 activation, resulted in significant reduction of STAT3 phosphorylation. Moreover, knockdown of TRiC in HEPG2 cells reduced their sensitivity to IL-6 induced STAT3 activation. There is some evidence to suggest that other STAT proteins also may depend on TRiC for function. For example, we have shown that STAT1 known for its pivotal role in tumor immune surveillance also interacts with TRiC in rabbit reticulocyte lysates [87]. Thus, in addition to HSP chaperones, TRiC chaperonin contributes to carcinogenesis through its contribution to STAT3 signaling.

### 4.3. Interaction of TRiC with p53 Promotes the Protein Folding and Activity of p53

p53 is a well-studied tumor suppressor protein that plays a critical role in preventing malignant cancer cell development [94]. p53 primarily functions as a transcription factor that modulates the expression of a variety of genes involved in cellular responses such as cell-cycle arrest and apoptosis [95]. As the activities of p53 must be strictly controlled to guide normal growth and development, numerous mechanisms to regulate p53 activity have been revealed, including translational control, protein stability, subcellular localization, and interaction with other transcriptional co-factors [96]. Mutations of p53 are among the most common mutations found in tumors. Most p53 mutations are single residue missense mutations that occur predominantly within the central DNA binding domain, some of which result in protein misfolding [97]. The protein stability of wild type and mutant p53 has been demonstrated to be regulated by p53 binding to HSP70, HSP90, and TRiC [98,99]. In particular, Vousden’s group [12] demonstrated that TRiC binds and promotes the protein folding of p53 by the direct interaction with the N-terminus of p53. The consequence of loss or modulation of this interaction results in the accumulation of misfolded p53, which can promote cancer development similar to mutant p53. They found enhanced invasive growth with depletion of TRiC in wild-type p53 cells, which is a phenotype similar to that observed in cells containing mutations, R175H and R273H, that causes p53 to misfold. However, they also observed that down-regulation of TRiC in cells containing the R273H mutant p53 inhibited the cells invasiveness, indicating that the contribution of TRiC to mutant p53-mediated oncogenesis is not as straightforward as it is for STAT3.

### 4.4. TRiC Modulates Cell Cycle Regulatory Proteins

Cell cycle deregulation is a salient feature of cancer. Accumulating evidence suggests that TRiC plays a critical role in the regulation of cell cycle progression. TRiC expression is strongly upregulated during cell growth, and assists in the folding of actin as well as other proteins required for cell growth [100]. It has been shown that CDC20 is dependent on TRiC for proper folding and incorporation into the multi-component E3 ligase Anaphase Promoting complex (APC) [100]. CDC20 is a highly conserved activator of the APC, required for cell cycle progression [101,102]. CDC20 contains several WD40 repeats that are known to bind TRiC; this domain architecture is common among adaptor proteins and helps mediate a diverse array of protein-protein interactions [51]. CDC20 is thought to selectively bind and recruit specific substrates to the APC complex for ubiquitination and subsequent destruction by the proteasome [103]. Major targets for CDC20 recruitment to the E3 ligase include critical cell cycle regulators, such as cyclinA, cyclinB1, p21 and securin. In addition, CDC20 is known to modulate key anti-apoptotic proteins MCL1 and BIM suggesting an important role in the regulation of apoptosis [104,105]. Thus, it is not surprising that several studies have shown that CDC20 may function as an oncoprotein to promote the development and progression of human cancers [106]. Elevated levels of CDC20 have been found in a wide variety of cancers. Moreover, elimination or blocking of CDC20 in several cancer models results in mitotic arrest and apoptosis [107,108]. Others studies show that CDC20 may have a role in genetic instability as it targets histone demethylases and some key members of the DNA damage repair pathway [109,110].

Cell cycle progression in proliferating cells is driven through the activity of cyclin-dependent kinases. TRiC has been known to modulate cyclin activity; for instance, maturation of cyclin E requires TRiC activity and biochemical studies in yeast and human cells show that TRiC associates with nascent cyclin E and assists in its folding and assembly with Cdk2 [11]. The control of cell cycle progression is achieved by the precise manipulation of the conformational stability of these kinases requiring rapid temporal degradation via the ubiquitin proteasome system. Abnormal stability of cycling proteins is known to lead to dysregulation of cell cycle progression. Several studies show that the stringent control of cyclin E associated with normal cells is disrupted in cancer [111]. There is a strong correlation between deregulated expression of cyclin E and aggressive tumor phenotypes. In addition to cyclin E, TRiC also has been implicated in the biogenesis of other kinases involved in cell cycle regulation; for example, TRiC is required for functional biogenesis of PLK1 whose increased activity is known to override DNA damage repair and promote genetic instability [112,113]. Taken together, TRiC contributes to the folding and activity of cell cycle regulators and its deregulation is likely to link to cancer cell progression.

### 4.5. Contribution of LOX-1, a Newly Identified TRiC Substrate, to Inflammation and Oncogenesis

LOX-1 is emerging as an oncoprotein due to its contribution to malignant transformation in the setting of inflammation [114]. Several clinical and epidemiological studies indicate a role for chronic inflammation in cancer development [21]; LOX-1 is a link between chronic inflammation and cancer [115,116]. LOX-1 binds oxidized low-density lipoprotein (OxLDL) inducing a chronic inflammatory signal implicated in atherosclerosis [117,118,119] and, perhaps cancer.

We recently demonstrated that LOX-1 binds directly to TRiC in endothelial cells and that treatment of cells with OxLDL causes disassociation of the LOX-1/TRiC complex [120]. TRiC also is required for the assembly and function of a complex that includes silencing mediator for retinoid or thyroid hormone (SMRT) and histone deacetylase (HDAC) [121]. In addition, TRiC modulates the activity of histone acetyltransferase (HAT), although it is not clear if the folding function of TRiC protein contributes to this process [122]. HDAC and HAT counteractively regulate acetylation of histone, which is a primary epigenetic determinant of gene activity. HATs and HDACs have been associated with B- and T-cell malignancies and several HAT and HDAC inhibitors are currently under clinical investigation [123].

OxLDL and LOX-1 may induce malignant transformation by activating NF-κB [114]. NF-κB is a master transcriptional regulator of inflammation; it induces expression of large number of pro-inflammatory, as well as pro-oncogenic, genes. Epigenetic regulation of genes by HDAC and HAT allows access of transcription factors, such as NF-κB, to gene promoter regions; thus, dysregulation in HAT and HDAC activity through altered TRiC levels or activity may contribute to increased NF-κB activity and cancer.

## 5. Summary

It is clear that chaperones are critical mediators of oncogenesis. In this review, we provide a brief introductory overview on the role of HSPs in cancer before focusing on the chaperonin TRiC. We discuss how TRIC activity is linked to oncogenesis through its clients—oncoproteins and tumor suppressor proteins—that have well-established roles in cancer. The examples we highlight indicate that TRiC may play an important role in oncogenesis by modulating cancer cell growth, apoptosis, and genome instability. Major advances have been made in clarifying how TRiC folds its substrates, but additional work is necessary to elucidate the mechanisms of TRiC substrate recognition, which could be exploited to develop strategies for identifying new cancer therapies. In addition, because TRiC is required for the proper folding of ~10% of the proteome, a systematic approach to the identification of TRiC substrates that contribute to the many different types of cancer will be challenging Thus, development of new methodologies and further experimental studies are warranted to increase our understanding of the role TRiC in cancer development and progression.
